# Constructing a New Biomass‐Based Bistatic Window for Solar Regulation

**DOI:** 10.1002/advs.202401991

**Published:** 2024-05-29

**Authors:** Jihong Pu, Miao Han, Chao Shen, Julian Wang, Lin Lu

**Affiliations:** ^1^ Department of Building Environment and Energy Engineering The Hong Kong Polytechnic University Hong Kong 100872 China; ^2^ School of Architecture and Design Harbin Institute of Technology Key Laboratory of Cold Region Urban and Rural Human Settlement Environment Science and Technology Ministry of Industry and Information Technology Harbin 150090 China; ^3^ Department of Architectural Engineering Pennsylvania State University University Park PA 16802 USA

**Keywords:** bio‐massed material, bistatic, low‐carbon buildings, solar regulation

## Abstract

Smart windows effectively respond to the ever‐changing climatic conditions, offering a smart solution for low‐carbon buildings. However, current smart windows derived from chromic materials often have inferior solar modulation ability, or showcase high haze that obstructs outdoor views. Here, instead of developing new chromic materials, a new bistatic window is proposed for ultra‐high solar modulation and luminous transmission. The new developed window can reduce the indoor surface temperature for ≈11 °C, and reduce the building space cooling and heating energy consumption by 30% to 40%, providing significant energy‐related advances over traditional smart windows. In detail, the bistatic window exhibits excellent solar modulation ability (ΔT_sol_ = 61%), high visible transmittance in both bleached (***T*_lum,bleached_** = 91%) and colored (***T*_lum,colored_** = 56%) states, low haze (< 1%), rapid switching response (switching time < 1 min), high color rendering index (CRI > 80), and long‐cyclic stability after 1000 cycles. With the advantages of facile fabrication and scalability, it is foreseen the developed bistatic window holds promising prospect for the next‐generation low‐carbon buildings, paving a new way for future advancements in the fields of smart windows.

## Graphical abstract

A dual‐mode bistatic window is developed, which can achieve transparency switching through a 180‐degree rotation. It exhibits outstanding solar modulation ability (ΔT_sol_ = 61%), as well as high visible transmittance in both bleached (***T*_lum,bleached_** = 91%) and colored (***T*_lum,colored_** = 56%) states, offering a new approach for solar regulation in buildings.

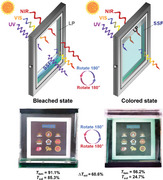

## 1 Introduction

Buildings contribute to 36% of total energy consumption and 37% of global carbon emissions^.[^ ^1^ ^]^ Windows are the weakest link for building energy‐saving, which approximately account for 40% of building energy loss. Thus, energy‐saving innovation in windows has become a crucial approach to achieve carbon neutrality in buildings. Generally, energy‐saving improvements for windows are focused on reducing the U‐value and controlling the solar transmittance. Thermal insulation technologies, such as vacuum windows, multi‐layered low‐e windows,^[^ ^2^ ^]^ liquid‐flow windows,^[^ ^3^ ^]^ and aerogel‐based windows,^[^ ^4^ ^]^ are fairly mature and commercially available, which can decrease the overall U‐value to below 1.0 W m^−2^ K^−1^. To further improve the energy‐efficiency of windows in varying climatic conditions, it is crucial to regulate the solar transmittance.

Dynamic smart windows enable buildings with tunable solar transmittance for hot and cold seasons,^[^ ^5^ ^]^ and they can be categorized into passive smart windows and active smart windows. Passive smart windows featuring self‐triggered spectrum regulating ability, including photochromic (***PC***),^[^ ^6^, ^7^ ^]^ humidity‐responsive smart windows,^[^ ^8^ ^]^ and thermochromic (***TC***),^[^ ^9^, ^10^, ^11^, ^12^ ^]^ have been sought after in various applications. ***TC*** is the most desirable way for transparency‐switching trigger and VO_2_ has gained recognition as a promising material for ***TC*** application.^[^ ^13^, ^14^ ^]^ However, with the limitations in effectively transmitting short‐wave visible light, VO_2_ may cause side effects such as narrow color gamut and non‐visual defects.^[^ ^15^, ^16^, ^17^ ^]^ In addition, the VO_2_‐based glazings show inferior photopic luminous transmittance (***T*_lum_**) and solar modulation ability (**Δ*T*_sol_**). Even the state‐of‐the‐art VO_2_ smart windows cannot achieve a ***T*_lum_** of 50% and a **Δ*T*_sol_** above 30% simultaneously.^[^ ^13^, ^18^, ^19^ ^]^ The hydrogel‐based windows achieve transparency‐switching through tuning the scattering behaviors of temperature‐responsive hydrogel, and they are highly transparent in the cold state and possess excellent solar modulation capabilities, exceeding 70%. However, their scattering‐based solar modulation comes a high level of haze and undesired opacity,^[^ ^20^, ^21^, ^22^, ^23^, ^24^ ^]^ and their luminous transmittance in opaque status is uncompetitive.

Active smart windows with external stimulated optical regulation, including magnetochromic,^[^ ^25^ ^]^ mechanochromic,^[^ ^26^ ^]^ electrochromic (***EC***),^[^ ^27^, ^28^ ^]^ polymer‐dispersed liquid crystals (***PDLC***),^[^ ^29^ ^]^ and suspended particle devices (***SPD***),^[^ ^30^ ^]^ provide high level of controllability and are more commercially available. ***EC*** devices, particularly the WO_3_‐ and V_2_O_5_‐ based products,^[^ ^31^ ^]^ are reliable and flexible techniques that have witnessed streamlined production. However, ***EC*** devices are primarily targeted to regulate visible light, and the tenability in solar ***NIR*** region is limited. Additionally, most of the ***EC*** windows cannot offer efficient control across the entire visible spectrum, leading to a narrow color gamut.^[^ ^32^ ^]^ As a result, although great progress has been achieved in smart windows, neither ***TC*** nor ***EC*** smart windows are ready for scalable residential use at this stage. For the current dynamic optical materials used in smart windows, the inherent issues of irreversible switching, poor luminous level, high scattering, and low solar modulation ability still remain to be settled.

**Figure 1:**
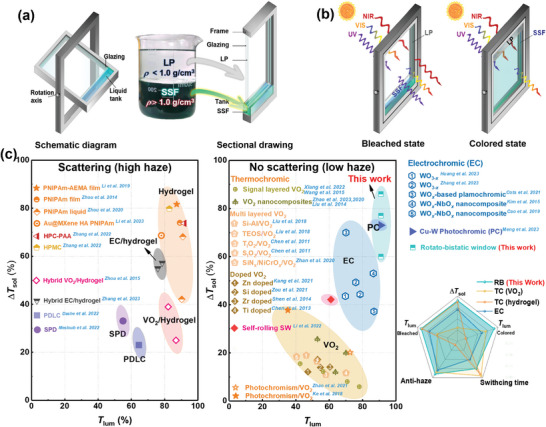
a) Schematic representation of the **RB** window; b) working principle of the **RB** window; c) performance comparison of the **RB** window and its counterparts (**TC**, **EC**, **PC**, **PDLC,** and **SPD** windows).

Here, instead of developing new chromic materials, we propose a new concept for active solar regulation in windows. This concept involves the synergistic development of two static optical materials and a reversible structure (**Figure** **1a**). The resulting window operates in dual mode, through switching the optical fluids between two glazings, as shown in Figure 1b. The optical switching is achieved through 180‐degree rotation of the window, herein referred as rotatable‐bistatic (**RB**) window. Videos S1 and S2 (Supporting Information) show the transparency switching processes. Before this study, it should be mentioned the 360‐degree rotatable window is already commercially available, which is specifically designed to facilitate glazing cleaning. This work takes the rotatable window a step further by incorporating tunable optical transmittance. The developed **RB** window demonstrates high visible transmittances in both bleached state (***T*_lum_ = 91%**) and colored state (***T*_lum_ = 56%**). It also exhibits excellent solar modulation ability (Δ ***T*_sol_ = 61%**), fast transparency switching, and long‐cyclic stability, offering significant advances over other state‐of‐the‐art smart windows such as the ***TC***,^[^ ^10^, ^19^, ^20^, ^21^, ^22^, ^33^, ^34^, ^35^, ^36^, ^37^, ^38^, ^39^, ^40^, ^41^, ^42^, ^43^, ^44^, ^45^, ^46^ ^]^ ***EC***,^[^ ^27^, ^47^, ^48^, ^49^, ^50^ ^]^ **PC**,^[^ ^7^ ^]^ **PDLC**,^[^ ^29^ ^]^ and **SPD**‐based^[^ ^30^ ^]^ windows (Figure 1c, detailed comparison data provided in Table S1, Supporting Information). With facile manufacturing and scalability, the **RB** window is a promising candidate for the next‐generation green buildings.

## 2 Material and Structural Design of the RB Window

### 2.1 Ideal Optical Properties of Window

**Figure 2:**
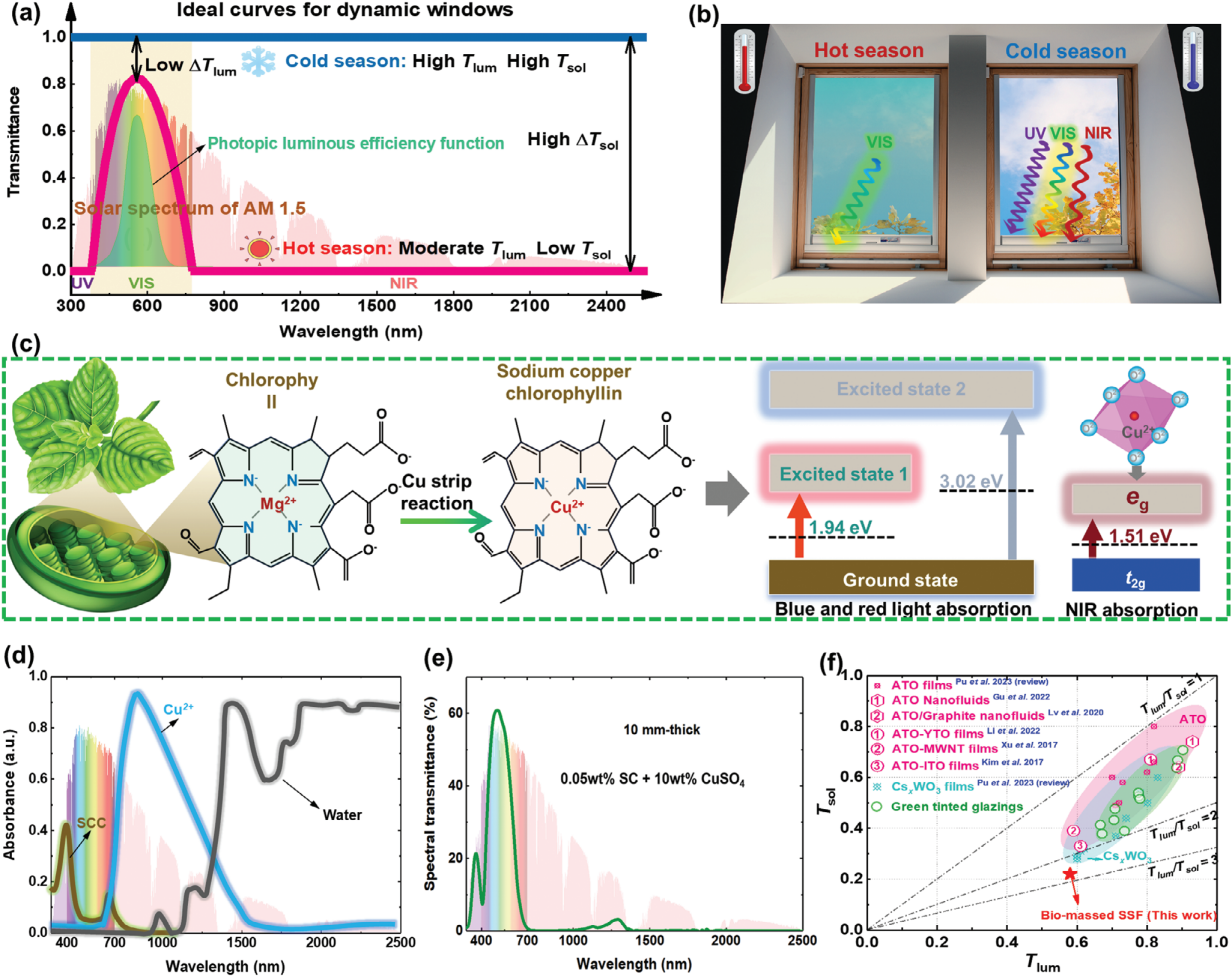
a) Ideal transmittance curves for dynamic windows in hot and cold seasons; b) schematic representation of the ideal windows for hot and cold seasons; **c)** schematic of the preparing process of **SC**, and the band‐gap widths of **SC** and Cu^2+^; d) solar absorption features of **SC**/Cu^2+^ hybrid solution; e) spectral transmittance of the prepared **SC**/Cu^2+^ hybrid solution; f) comparison of the performance for the biomass SSF developed in this study and other counterparts.^[^ ^51^ ^]^

The ideal optical properties for window is clearly seasonal. As shown in **Figure** **2a**, in cold seasons, an ideal window should be fully transparent across the entire solar spectrum, to maximize the solar heat gain through window structure in buildings. Conversely, in hot seasons, windows are supposed to minimize the indoor solar heat gain, and remain high ***VIS*** transparency for efficient daylight. To adapt to hot seasons, the optimal solution is to use spectrally selective designs that block the entire **NIR** spectrum and selectively block certain portion of red and blue wavelengths within the **VIS** spectrum. These designs prioritize maximum transparency in the green **VIS** light band, as depicted in Figure 2a. As a result, the windows exhibit a light green color, as shown in Figure 2b. By prioritizing the transmission of green light, the window will achieve an ultra‐high light‐to‐solar heat gain (**LSG**) that surpasses the performance of state‐of‐the‐art spectrally selective materials.^[^ ^51^ ^]^

### 2.2 Material Design for the Ideal Window

In addition to high ***LSG***, the natural green color has been proven to be visually soothing for the human eye, and it is also associated with various healthy photo‐biological benefits for dwellers.^[^ ^52^, ^53^ ^]^ Inspired by the natural green color from in foliage, a novel bio‐massed spectrally selective fluids (**SSF**), that replicates the color found in natural plants, was developed to match the windows’ ideal optical property in hot season. First, a reaction involving a copper strip was conducted with chlorophyll, to replace Mg^2+^ with Cu^2+^ (Figure 2c), thereby forming the stable and water‐soluble sodium copper chlorophyllin (**SC**). Then, to further enhance the ***NIR*** absorption capabilities, excessive CuSO_4_ was introduced to form the **SC**/CuSO_4_ hybrid aqueous solution. Meanwhile, polysorbate‐80, which helps prevent the aggregation of **SC** in acid environment, was added to enhance the stability of the **SC**/CuSO_4_ hybrid solution.

The resulting **SSF** brings together the extinction characteristics of **SC**, [CuO_6_], and water molecules. The absorption of **SC** peaks at 405 nm and 640 nm, corresponding to the bandgap widths of 3.02 eV and 1.94 eV (Figure 2c), respectively. These absorption peaks allow for the partial filtering out of certain blue‐ and red‐light wavelengths. In addition, the ***d‐d*** transition of [CuO_6_] causes the absorption ≈820 nm, further enhancing the overall **NIR** absorption capabilities of the hybrid solution. Furthermore, the water molecules can block the long‐wave **NIR** with wavelength greater than 1200 nm. This additional absorption feature ensures that the sunlight is converted into an ideal “cold light source”, as depicted in Figure 2d,e. Comparing the performance of the new biomass **SSF** with other solar control materials recorded in a previous study^[^ ^51^ ^]^ and the green dyes used in windows (Table S2, Supporting Information), the proposed **SSF** stands out with the highest ***T*_lum_**/***T*_sol_** value of around 3.0 (Figure 2f), representing significant energy‐related advances over other state‐of‐the‐art solar control materials.^[^ ^51^ ^]^ In addition, the prepared liquid was demonstrated to have a fairly stable optical performance, as the aging test indicated it could maintain a constant optical transmittance for a period over 8 months (Figures S1 and S2, Supporting Information). The **UV** experiments also demonstrated the prepared liquid has an excellent **UV** stability, as indicated in Figure S3 (Supporting Information).

### 2.3 Structural Design of the RB Window

A counter fluids to the **SSF**, i.e., the liquid paraffin (**LP**), was employed in the **RB** (Figure 1a) window. The **LP** and the **SSF** are immiscible and act as two distinct mediums, providing two different optical functionalities for the window. The switching between these two fluids is enabled by the unique 180‐degree rotatable structural design, as seen from Figure 1a. The **SSF** and **LP** have different density (Figure S4, Supporting Information). In bleached state, the liquid tank is positioned at the bottom of the window, the gravity causes the **SSF** to sink into the liquid tank, while the **LP** rises to occupy the hollow gap. This configuration yields a highly transparent window, allowing for maximum solar heat gain. Conversely, in hot conditions, the liquid tank is relocated to the top position through 180‐degree rotation, causing the **LP** to float into the tank while the **SSF** sinks into the hollow gap. As a result, the window exhibits the optical properties of the **SSF**, characterized by high luminous transmittance and low solar heat gain. Figure S5 (Supporting Information) demonstrates the weathering resistance performance of the **RB** structure, indicating that the device can maintain a consistently stable optical switching ability even after a duration of 9 months. This characteristic allows for prolonged and extended use of the device.

## 3 Physical Properties of the RB Window

### 3.1 Optical Properties

**Figure 3:**
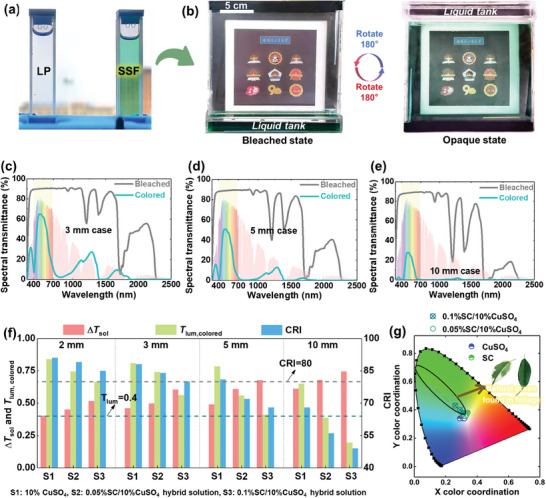
a) The optical photo of the two optical fluids; b) optical photos of the **RB** window in bleached and colored states; c) transmittance spectra for3 mm‐, d) 5 mm‐ and e) 10 mm‐thick **RB** windows; f) performance comparisons of the **RB** windows with different thickness and different **SSF**; g) chromaticity diagram of the **RB** window with different kinds of **SSF**.

The **LP** and **SSF** exhibit high optical contrast (**Figure** **3a**), as the **LP** possesses excellent transparency across the solar spectrum, while the **SSF** displays a distinct spectral selectivity. Figure 3b gives the optical photographs of a 20 × 20 mm^2^ **RB** window sample, with the 0.1%SC/10%CuSO_4_ hybrid solution. The sample offers transparency in both of these two states. In bleached state, the sample shows high transparency and color neutralization. In colored state, the color closely aligns with the ideal windows for hot seasons (Figure 2b), with a high transparency and light green color.

Figure 3c–e provide comparisons of the spectral transmittance of **RB** windows with different hollow thicknesses, utilizing a 0.1%SC/10%CuSO_4_ hybrid solution as the **SSF**. In the bleached state, the **RB** windows have high transmittance across the entire visible spectrum, with an overall photopic visible transmittances around 91%, and high solar transmittance above 85% (Table S3, Supporting Information). While in colored states, the solar transmittances of these three samples reduced ≈60.6%, 67.6%, and 74.6% respectively, indicating the solar modulation ability of the **RB** windows is higher than most of the **TC** and **EC** windows. Additionally, the **RB** windows offer reasonable luminous transmittances in colored status (Table S3, Supporting Information), which demonstrate the optical‐advantages of **RB** windows over other smart windows.^[^ ^20^, ^21^, ^22^ ^]^

For further parametric optimization, the optical transmittance of samples with different thicknesses and different SC and CuSO_4_ concentration were tested for analysis (Figure S6, Supporting Information), and the corresponding parameters, include the visible transmittances, solar transmittances, melanopic transmittances, solar heat gain coefficient (**SHGC**) and color rendering index (**CRI**) are recorded in Table S3 (Supporting Information). Figure 3f compares the performance of **RB** windows in terms of solar modulation ability, visible transmittance, and **CRI** in the colored state. Evidently, increased fluids thickness and **SC**’s concentration improve the solar modulation ability, but the improvements are accompanied by a decreased visible transmittance and **CRI**. For residential building applications, the visual property with **CRI** above 80, and visible transmittance above 0.4 is essential for creating an appropriate light environment in buildings.^[^ ^51^ ^]^ Based on the comparisons shown in Figure 3f, the 0.1%SC/10%CuSO_4_, which appears a light‐green color (Figure 3g), is recommended, as it effectively addresses the trade‐off between solar heat modulation ability and daylight quality. In detail, this configuration shows a high solar modulation ability of 60.6%, excellent visible transmittances of 90% and 56.2% in bleached and colored status respectively, and a feasible **CRI** above 80 in the colored state (Table S3, Supporting Information). Furthermore, the melanopic transmittance of the 0.1%SC/10%CuSO_4_ solution is approximately equal to its visible transmittance. The calculated Circadian Action Factor (***CAF***) is ≈1 (Table S3, Supporting Information), indicating that the solution can provide a favorable photo‐biological performance similar to natural lighting.

### 3.2 Fluid Dynamics Behaviors

The **RB** window achieves optical switching via the gravity‐driven flow of **SSF** and **LP**. The density difference between **SSF** and **LP** causes these two fluids to separate, forming distinct layers. Videos S1 and S2 (Supporting Information) show the coloring and bleaching processes of a **RB** window sample with dimensions of 20 cm (height) × 20 cm (length), respectively. Meanwhile, the photos of the sample at different stages were captured and presented in Figure S7 (Supporting Information), and Figure S8 (Supporting Information) further shows the variation in the visible transmittance of the top and bottom points. The results indicate that it takes ≈10 s for **SSF** to flow from the top to the bottom position in the coloring process, while the bleaching process takes ≈7 s. To explore more details in fluid diffusion and mixing processes, **CFD** simulations was conducted based on the volume of fluid (**VOF**) model. Figure S9 (Supporting Information) shows the visual results of the simulation, which indicates that the coloring process for the 20 cm × 20 cm **RB** window sample takes ≈20 s, while the bleaching process takes ≈16 s.

**Figure 4:**
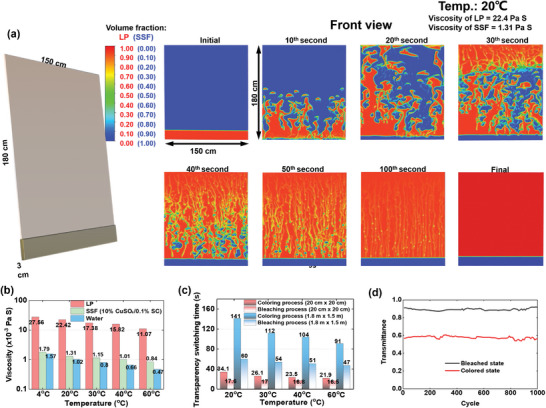
a) CFD simulation results of the fluids switching process; b) comparisons of the viscosity at different temperature; c) transparency‐switching time at different temperature conditions, d) cyclic test of the **RB** window.

To clarify the transparency‐switching for the real scaled **RB** window, the **CFD** simulation was conducted in a **RB** window with dimensions of 150 cm (length) × 180 cm (height). **Figure** **4a** shows the bleaching process at a temperature of 20 °C. Temperature of fluid will affect its viscosity, and thereby affect the transparency switching process. Figure 4b presents the measured viscosity of SSF and LP under different temperature conditions. Further, Figure 4c shows the simulated transparency switching time of the **RB** windows, for both the coloring process and the bleaching process. A smaller window size and higher temperature results in a faster transparency switching. For the real‐scaled **RB** window (150 cm × 180 cm) at temperature of 20 to 60 °C, the coloring process requires 91 to 141 s, while the time consumption for bleaching process is smaller than 1 min. This result indicates that the **RB** window can achieve faster transparency switching than most of the **EC** windows,^[^ ^47^, ^48^, ^49^, ^50^ ^]^

Figure 4d displays the results of the cyclic test conducted on the 20 cm × 20 cm **RB** window sample at room temperature (around 24 °C). The visible transmittance of the **RB** window remains fairly constant in both the bleached and colored states, even after 1000 cycles, demonstrating the cyclic stability of the **RB** window during extended use. Considering its excellent solar modulation ability, long‐term stability, durability in optical performance, high visible transmittance, flexible transparency switching, and easy cleaning features, the **RB** window proves highly attractive for residential applications.

## 4 Energy‐Saving Demonstration of the RB Window

### 4.1 Experimental Demonstration of the Energy‐Saving Performance

**Figure 5:**
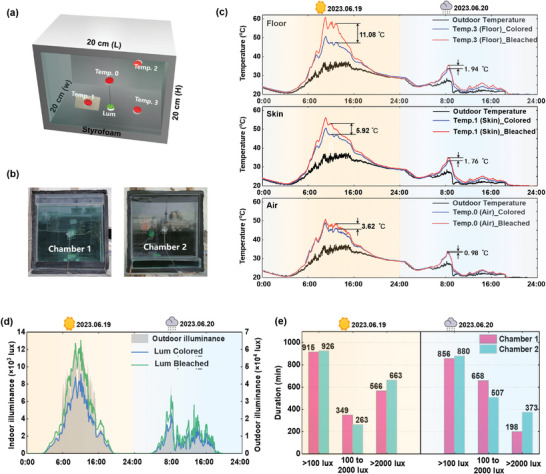
a) Layout of sensors placement in the chamber; b) experimental chambers with **RB** window in colored (left) and bleached states (right); c) comparisons of the temperatures of the chambers’ floor (above), artificial skin (middle) and indoor air (below); comparisons of the indoor illuminance d,e) durations of **UDI**_100‐2000_.

To further demonstrate the energy‐saving performance of the **RB** window, continuous outdoor experimental tests were conducted on two summer days in Harbin, specifically on June 19 (sunny day) and June 20 (rainy day), 2023 respectively. The experimental chambers have a dimension of 20 cm × 20 cm × 20 cm, with south surfaces installing the **RB** windows. The other surfaces of the chambers were spliced with 5 mm‐thick PMMA plates and wrapped with 1 cm‐thick polyethylene foam (**Figure** **5a**). In Chamber 1, the **RB** window was in the colored state, while in Chamber 2, the **RB** window remained in bleached state (Figure 5b) throughout the experiments. During the tests, two artificial skins were placed on the floor of the chambers, mimicking the solar absorption features of human skin (Figure S10, Supporting Information). Temperature and luminous sensors were strategically placed to continuously record data for further analysis, and detailed arrangement can be seen in Figure 5a.

The outdoor solar radiation levels of the two experimental days were recorded (Figure S11, Supporting Information). On the sunny day (19/06/2023), the maximum surface temperature of the inner glazing in Chamber 1 was ≈4.52 °C higher than Chamber 2, at the same time, the difference for the outer glazing surface was 5.64 °C (Figure S12, Supporting Information), indicating a secondary heat transfer caused by the solar absorption of window. Although it receives the secondary heat transfer from the inner glazing, the measured temperatures inside Chamber 1 were still lower than those of Chamber 2 (Figure 5c), and on the sunny day saw a more significant difference. On 19 June, Chamber 1 saw the highest maximum temperatures of 50.79 °C, 50.64 °C, and 49.09 °C for the chamber floor, artificial skin, and indoor air respectively, and these figures were ≈11.08 °C, 5.92 °C, and 3.62 °C higher in Chamber 2, demonstrating **RB** window's ability in regulating indoor solar heat gain.

Figure 5d shows the inside daylight at the floor of the chambers. On Jun 19, the floor of Chamber 2 showcased the highest illuminance level of 13 158 lux at 12:01 PM. In contrast, the illuminance level on the floor of Chamber 1 was 9154 lux at the same time. The useful daylight illuminance (UDI) recommends a desirable illuminance level between 100 lux to 2000 lux (U_100‐2000_), for effective daylighting in the work plane. As recorded on these two days (Figure 5e), both Chamber 1 and Chamber 2 maintained a similar duration of indoor illuminance above 100 lux, on both sunny days and rainy days. However, Chamber 1, with the **RB** window installed, achieved a longer duration within the U_100‐2000_. This is because the biomass dye in the **RB** window can effectively reduce glare. Comparing the data in Figure 5e, Chamber 1 has a duration of ≈349 min within the U_100‐2000_ on the sunny day, and 658 min on the rainy day. On the other hand, the duration for Chamber 2 is 263 and 507 min for the sunny day and rainy day, respectively. These results demonstrate that the **RB** window has the ability to create a more comfortable and suitable daylight environment, by mitigating glare and providing a more rational distribution of illuminance levels.

### 4.2 Energy‐Saving Simulation of the RB Window

**Figure 6:**
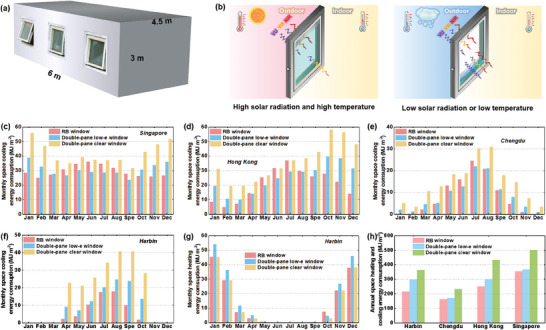
a) Building model used in the simulation, b) the working principles for the **RB** window; c–f) monthly space cooling energy consumption in different cities; g) monthly space heating energy consumption in Harbin; and h) the annual space cooling and heating energy consumption in these four cities.

To assess the energy‐saving of **RB** windows, simulations were conducted, considering an inner room of an office building, with dimensions of 6 m × 4.5 m × 3 m (**Figure** **6a**). The room has an external wall facing south, and other walls are internal walls. Three windows were installed on the south‐facing wall, each possessing identical dimensions of 1.8 m (length) × 1.5 m (height). The weather data collected in four cities different climatic conditions,^[^ ^54^, ^55^ ^]^ **i.e.,** Harbin, Hong Kong, Singapore, and Chengdu was used in the simulations. **RB** window with 3 mm‐gap and 0.1%SC/10%CuSO_4_ **SSF** was chosen as the object for simulation. The **RB** window was scheduled to be in colored state when there was a need for space cooling, and the solar radiation received by the window exceeded 200 W m^−2^. Otherwise, the windows remained in their bleached state (Figure 6b). For comparison, the same building models equipped with ordinary double‐pane low‐emissivity (low‐e) glazing windows and double‐pane clear windows were also simulated. Details regarding the window parameters, including the U‐value and **SHGC**, can be found in Table S4 (Supporting Information).

Figure 6c–f show the monthly energy consumption for building space cooling. Both **RB** window and double‐pane low‐e window could reduce space cooling energy consumption, compared to normal double‐pane clear windows. In the annual round, the **RB** window demonstrates a higher energy‐saving performance than the double‐pane low‐e window (Figure S13, Supporting Information). Figure 6g compares the monthly space heating consumption of different window types in Harbin. Low‐e window would passively reflect the solar radiation energy, negatively increasing the space heating load in cold seasons. While, the **RB** window can keep a high **SHGC** value in cold seasons by switching into the bleached state, which is positive for buildings to reduce the space heating energy consumption. In comparison to normal double layered clear window, the **RB** window can achieve an annual energy‐saving of 147 MJ m^−2^, 69 MJ m^−2^, 183 MJ m^−2^, 146 MJ m^−2^, in Harbin, Chengdu, Hong Kong, and Singapore, respectively, while this figure for the static low‐e window is 65.2 MJ m^−2^, 60.5 MJ m^−2^, 134 MJ m^−2^, 134 MJ m^−2^ respectively (Figure S13, Supporting Information; Figure 6h). These findings highlight the advantages of utilizing **RB** windows, in both hot and cold climatic zones, as it effectively addresses both space cooling and space heating requirements.

## 5 Conclusion

In this study, a novel **RB** window was designed for solar regulation of windows, meanwhile, a bio‐inspired spectrally selective material was proposed to effectively convert the solar spectrum into “cold light”. Compared with the traditional **TC** and **EC** windows, the demonstrated **RB** window showcases significant energy‐related advantages. In detail, the **RB** window presents superior solar modulation ability, surpassing 60%. It also exhibits high luminous transmittances in both bleached (90%) and colored states (56%). In addition, the window has high UV stability, excellent weathering assessment, low haze level (< 1%), rapid transparency switching, feasible color rendering index (**CRI** > 80), and rational photo‐biological effects. In the experimental demonstration of the **RB** window, the results indicate that the chamber with **RB** window could reduce its’ inner surface temperature by ≈11 °C on a sunny day, and it ensures a more rational indoor luminous level by effectively reducing the glare. In addition, simulations conducted in four cities demonstrated the feasibility of the **RB** window to both hot and cold regions, which can reduce the building space heating and cooling energy consumption of ≈30% to 40%. Based on these findings, the **RB** window technology provides a valuable solution for sustainable and environmentally conscious construction practices.

## Conflict of Interest

The authors declare no conflict of interest.

## Supporting information

Supporting Information

Supplemental Movie 1

Supplemental Movie 2

## Data Availability

The data that support the findings of this study are available from the corresponding author upon reasonable request.
