# Supplementary material for: Constructing a New Biomass‐Based Bistatic Window for Solar Regulation
Source: Adv Sci (Weinh). 2024 May 29;11(29):2401991. doi: 10.1002/advs.202401991 (PMC11304258; doi:10.1002/advs.202401991)
Supplement: Supplementary file 1 — Supporting Information [file ADVS-11-2401991-s002.docx]

Supporting Information

Constructing a New Biomass-based Bistatic Window for Solar Regulation

*Jihong Pu, Miao Han, Chao Shen*, Julian Wang, Lin Lu**

**Materials and methods**

*Materials*

The raw material of sodium copper chlorophyllin powder (standard: GB26687-2011; manufacture: Kirui Biotechnology Co., Ltd), copper sulfate (purity: 99.9%; manufacture: Xilong-Scientific), and Polysorbate-80 (manufacture: Shijieer) were used without further purification to prepare the **SSF**. The **LP** was purchased from Huangjia Biotechnology Co., Ltd., with the executive standards of Q/HHL01-98. The clear glazing and **PMMA** panes used in fabricating the RB window sample were provided by SAYS Co., Ltd, and their spectral transmittances are shown in **Figure S13**.

*Preparations of spectral splitting fluids*

Preparations of CuSO_4_ aqueous solution: the 10wt% CuSO_4_ aqueous solution was obtained by dissolving 100 g CuSO_4_·5H_2_O crystals into 900 ml DI water, with magnetic stirrer for 120 minutes at room temperature.

Preparations of SC aqueous solution: 120 g Polysorbate-80 (used as the dispersant for SC powder), and 177 ml DI water was mixed at 70 to 80 ℃ for 1 hour, and then 3 g SC powder was dissolved in the Polysorbate-80/water solution, with a magnetic stirrer for 120 minutes at room temperature. In such a process, the SC solution with a concentration of 1wt% was obtained. The 0.1wt% and 0.05wt% SC *aqueous* solution were obtained by dilutions of the 1wt% SC solution using DI water.

Preparations of CuSO_4_/SC aqueous solution: 100 g CuSO_4_·5H_2_O was dissolved in 400 ml DI water, with a magnetic stirrer for 120 minutes to obtain 20wt% CuSO_4_ solution, then 10 g SC powder with 120 g Polysorbate-80 was dissolved in 370 ml DI water to obtain 0.2wt% SC solution. The SC/CuSO_4_ blended solution with different concentrations was obtained by mixing the 20wt% CuSO_4_ solution, 0.2wt% SC solution, and DI water with different ratios.

*Fabrication of RB window sample*

The cuboid liquid tank was made of 3 mm-thick PMMA panes, with an outer dimension of 36 mm (width) × 26 mm (height) × 200 mm (length). Two clear glazing with dimensions of 200 mm (length) × 200 mm (height) × 3 mm (thick) connect the liquid tank. On the top surface of the liquid tank open a 3 mm × 200 mm slot that connects the gap between the two glazings. 120 ml **LP** and 120 ml **SSF** were infused in the liquid tank and the gap between the glazings, and then the **RB** window was sealed with cyanoacrylate adhesive.

*Characterization of the RB window*

The spectral transmittances of the fluids were tested by the Lambda-950 UV-VIS-NIR spectrophotometer (PerkinElmer, USA), using the cuvettes made of 3mm clear glass. With the spectral transmittances, the solar transmittances were calculated by **Equation (1)**; visible transmittances (***T*_lum_**) and melanopic transmittances (***T*_mel_**) were calculated by **Equation (2)**.

$T_{\text{sol}}=\frac{\int_{300}^{2500} T(\lambda)\Phi(\lambda)d\lambda}{\int_{300}^{2500} \Phi(\lambda)d\lambda}$ (1)

$T_{\text{lum}/\text{mel}}=\frac{\int_{380}^{780} T(\lambda)S_{\text{lum}/\text{mel}}(\lambda)\Phi(\lambda)d\lambda}{\int_{380}^{780} S_{\text{lum}/\text{mel}}(\lambda)\Phi(\lambda){}d\lambda}$ (2)

where ***T* (*λ*)** represents the spectral transmittance, **Φ (*λ*)** which is the solar irradiance spectra for air mass 1.5 (AM 1.5). *S***_lum_** **(*λ*)** and *S***_mel_** **(*λ*)** are the action spectra of photopic sensitivity and the action spectra of melanopic (ipRGC) sensitivity respectively.

$SHGC=\frac{A_{g}\cdot g+\alpha_{f}\cdot A_{f}\cdot\frac{U_{f}}{h_{f,out}}}{A_{w}}$ (3)

$g=T_{sol}+\alpha_{g}\cdot(\frac{\frac{1}{\frac{1}{h_{g,in}}+\frac{\sigma_{ssf}}{2\lambda_{ssf}}+\frac{\sigma_{g}}{\lambda_{g}}}}{\frac{1}{\frac{1}{h_{g,in}}+\frac{\sigma_{ssf}}{2\lambda_{ssf}}+\frac{\sigma_{g}}{\lambda_{g}}}+\frac{1}{\frac{1}{h_{g,out}}+\frac{\sigma_{ssf}}{2\lambda_{ssf}}+\frac{\sigma_{g}}{\lambda_{g}}}})$ (4)

where ***g*** is the ***g***-value of glazing system, which represents the combined effect of direct solar transmittance and the proportion of secondary heat transfer caused by solar absorbance of the **SSF**; ***α_f(g)_*** is the solar absorbance of window frame (glazing); ***U_f_*** is the overall heat transfer coefficient of the frame; ***σ_ssf(g)_*** represents the thickness of the **SSF** (glazing), ***λ_ssf(g)_*** represents the thermal conductivity of the **SSF** (glazing); ***h_g_,_in(out)_*** represents the convective heat transfer coefficients between indoor (outdoor) environment and the glazings’ inner (outer) surface, and it can be evaluated by:

$h_{a}=\left\{\begin{matrix} 2.8+3\cdot v_{a},ifv_{a}\leq5m/s \\ 6.15\cdot v_{a}^{0.8},ifv_{a}>5m/s \end{matrix}\right.$ (5)

where ***v_a_*** is the air flow velocity.

When calculating the **SHGC**, the thickness of **SSF** was considered as 3 mm, with a thermal conductivity of 0.694 W/(m·K), and the glazing thickness was 4 mm with a thermal conductivity of 1.2 W/(m·K). The outdoor air velocity was assumed to be approximately 3.0 m/s, according to the average outdoor air velocities for Hong Kong, Beijing, and Harbin were obtained from the Chinese Standard Weather Data (SCWD), which were 3.09 m/s, 3.08 m/s, and 2.35 m/s, respectively. The resulting SHGC values were calculated and are provided in **Table S3** of the supporting information.

The light-to-solar heat gain (***LSG***), is typically adopted by the U.S. National Fenestration Rating Councils (***NFRC***) and Department of Energy to evaluate the solar control ability of windows**^1^**, can be estimated by:

$LSG=\frac{T_{\text{lum}}}{SHGC}$ (6)

The circadian action factor, which is used to assess the effect of light on the circadian rhythm**^2^**, is calculated by:

$CAF=K\frac{\int_{380}^{780} I(\lambda)S_{\text{mel}}(\lambda)d\lambda}{\int_{380}^{780} I(\lambda)S_{\text{lum}}(\lambda)d\lambda}$ (7)

**
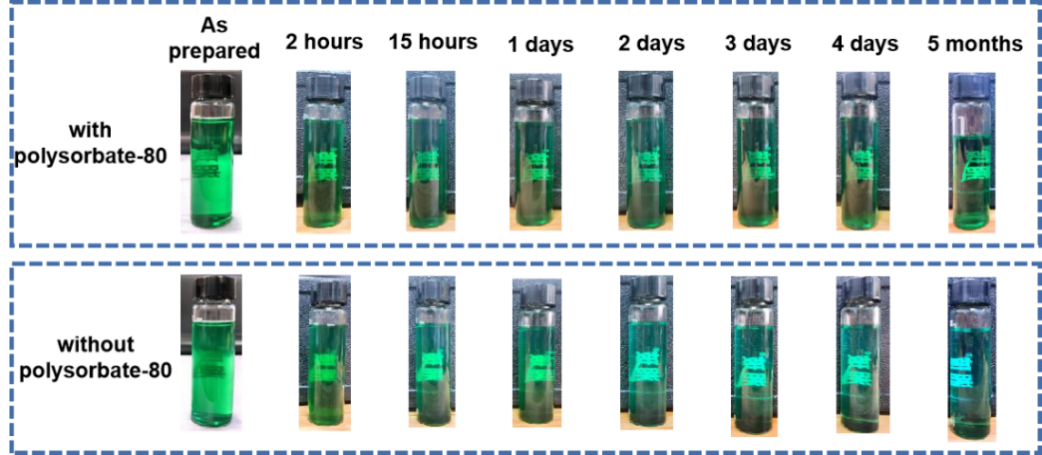
**

**Figure S1** Optical photos of the 0.1%SC/10%CuSO_4_ solution with (upper) and without (below) polysorbate-80 at different aging time.

|  |  |
| --- | --- |

**Figure S2** Spectral transmittance of 0.05%SC and 10%CuSO_4_ solution of different aging time.


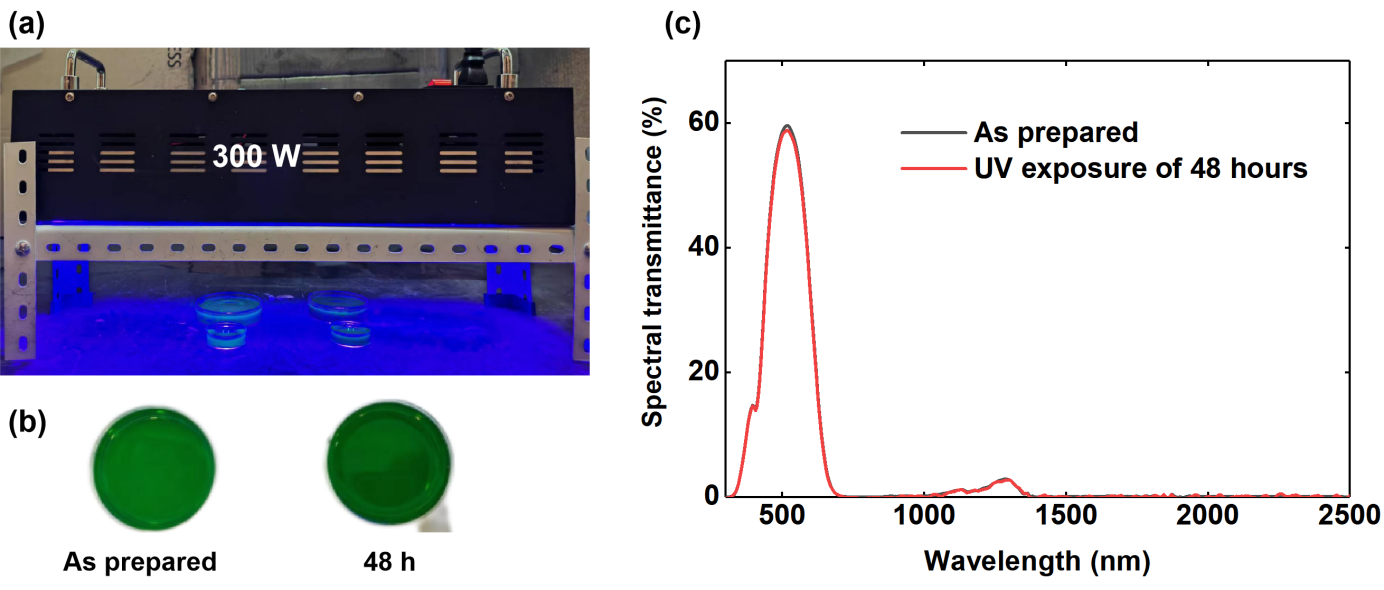


**Figure S3** (a) experimental rig for UV stability test, (b) optical photos of the 0.05%SC and 10%CuSO_4_ solutions; (c) results of the UV stability test.

**Figure S4** Comparisons of the density of the **LP** and **SPF**s.


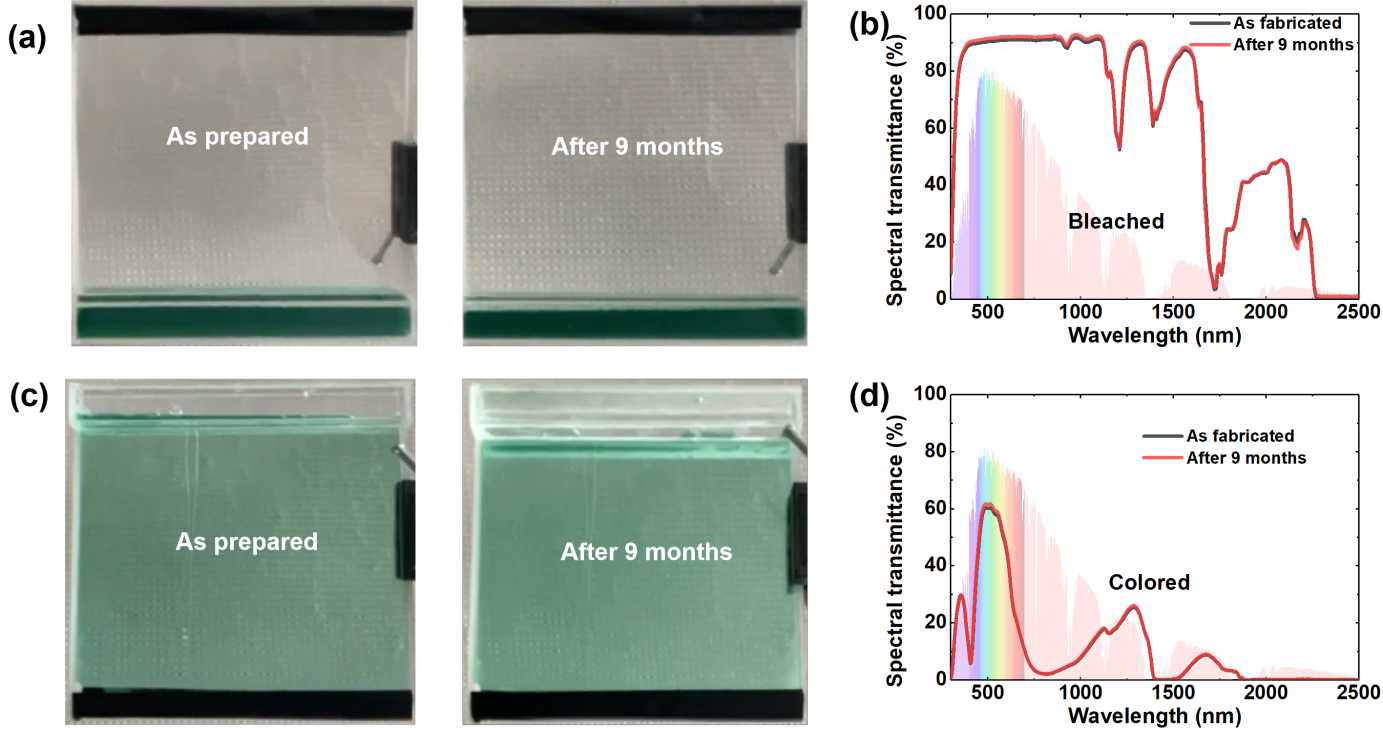


**Figure S5** (a) Optical photos of the bleached **RB** window with different aging time; (b) spectral transmittance of the bleached **RB** window with different aging time; (c) optical photos of the colored **RB** window with different aging time; and (d) spectral transmittance of the colored **RB** window with different aging time.

**
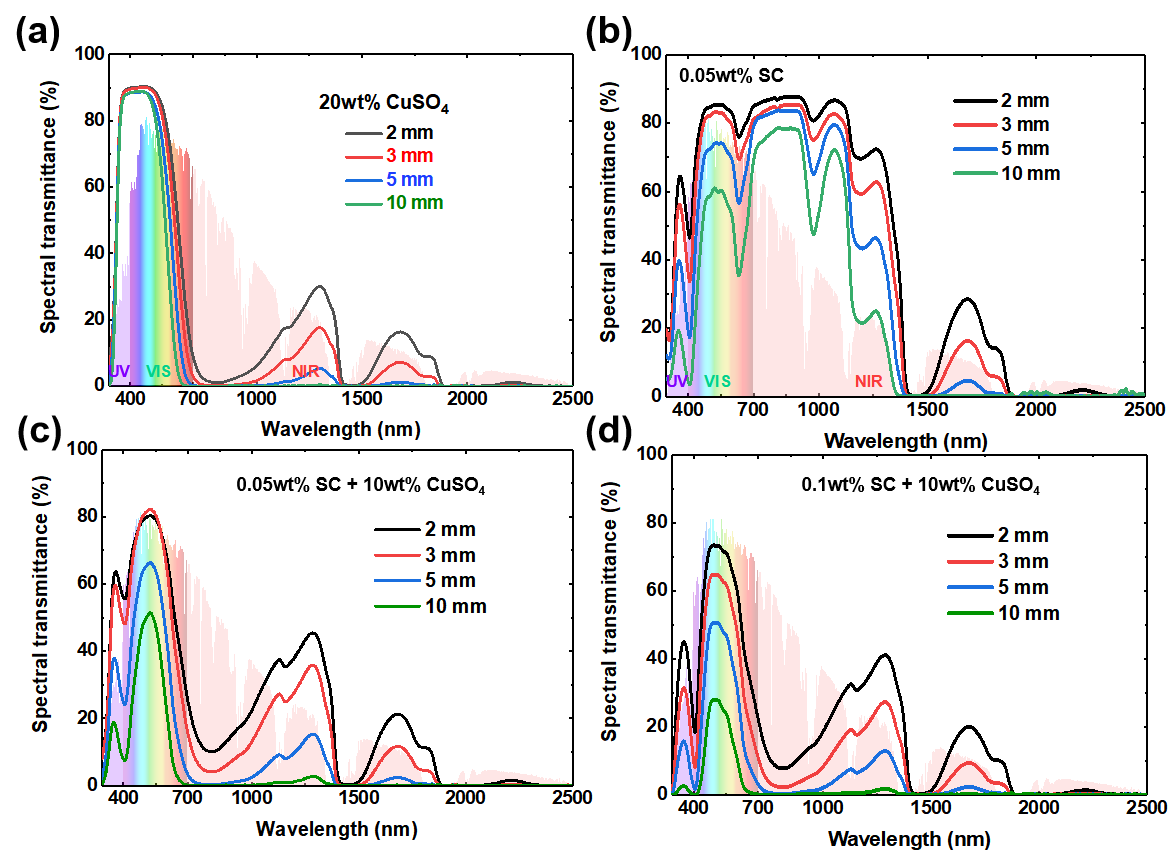
**

**Figure S6** Spectral transmittance of different **SSF** with different thickness: (a) 20%CuSO_4_ solution, (b) 0.05% SC solution, (c) 0.05%SC/10%CuSO_4_ solution, (d) 0.1%SC/10%CuSO_4_ solution.


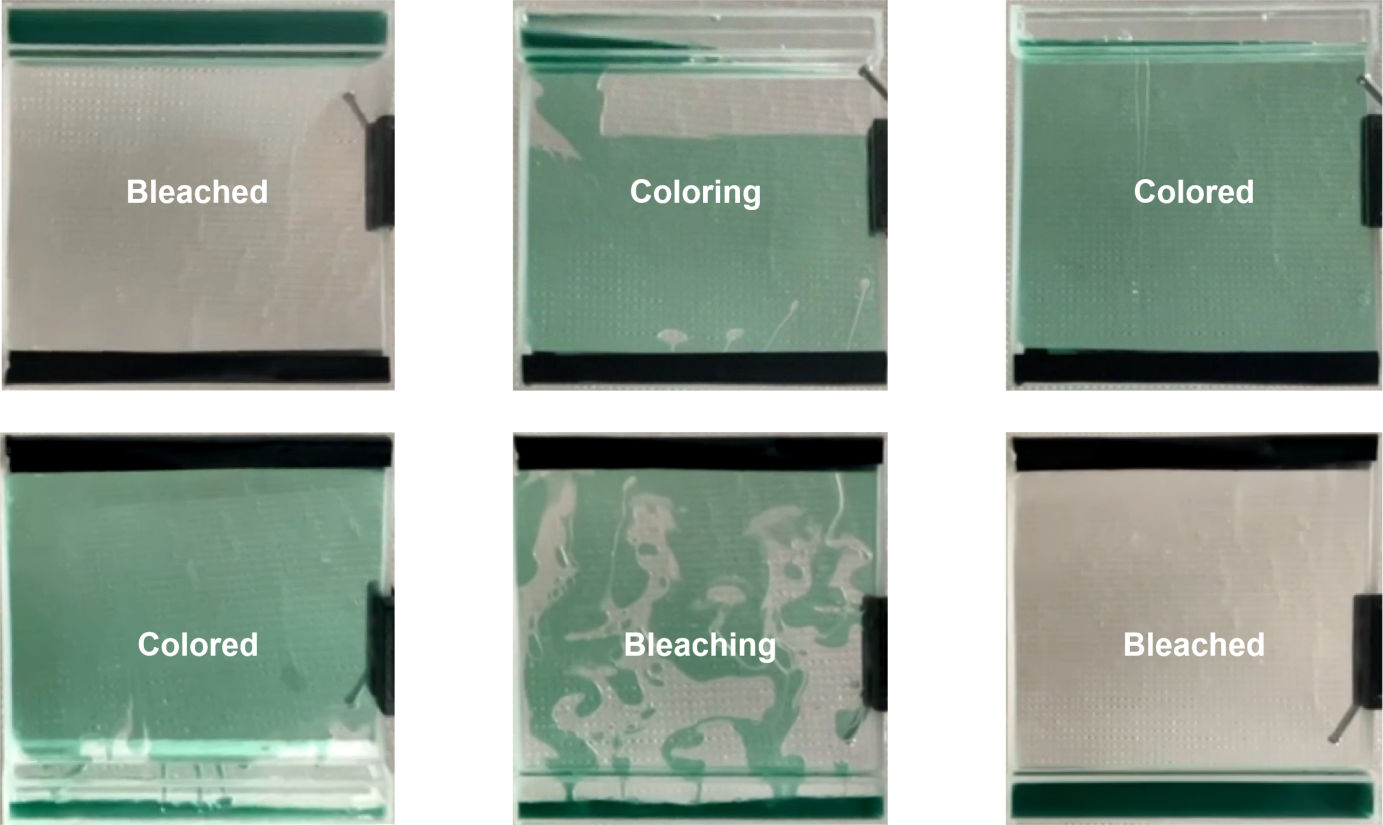


**Figure S7** Optical photos of the switching process


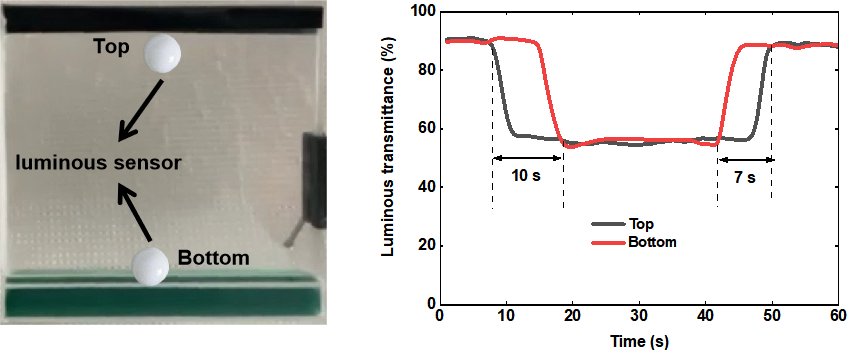


**Figure S8** Transparency change of specific points on the **RB** window sample.


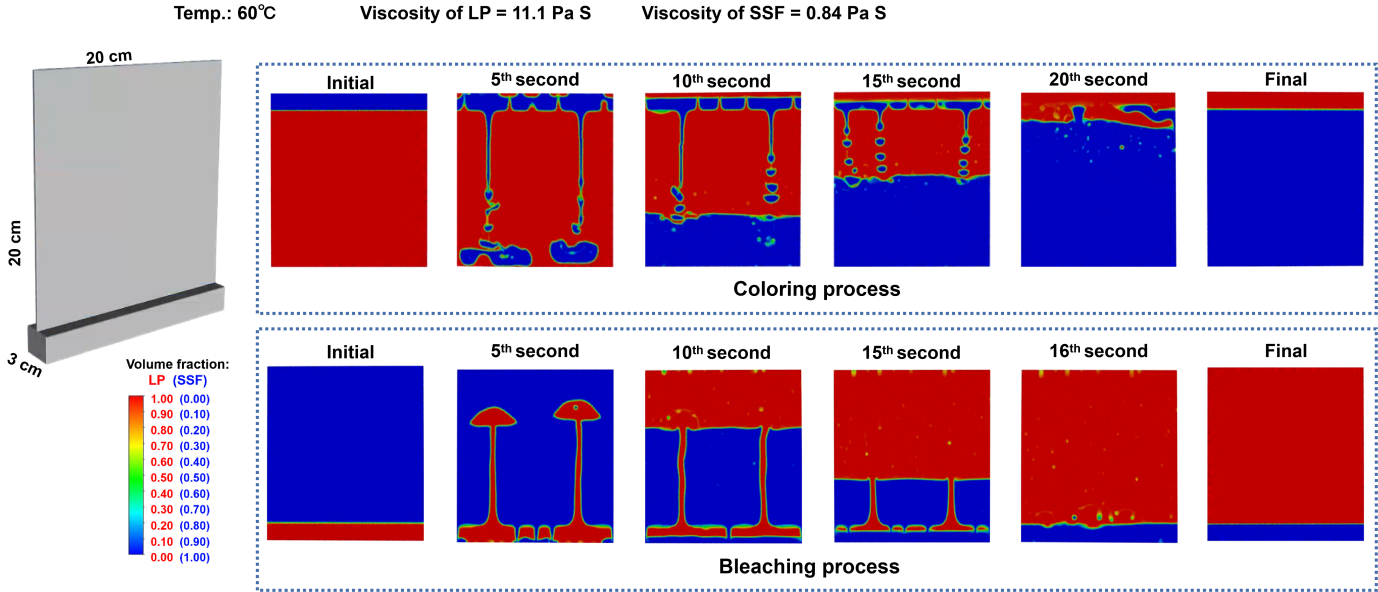


**Figure S9 CFD** simulation of the coloring and bleaching process of the **RB** window sample.


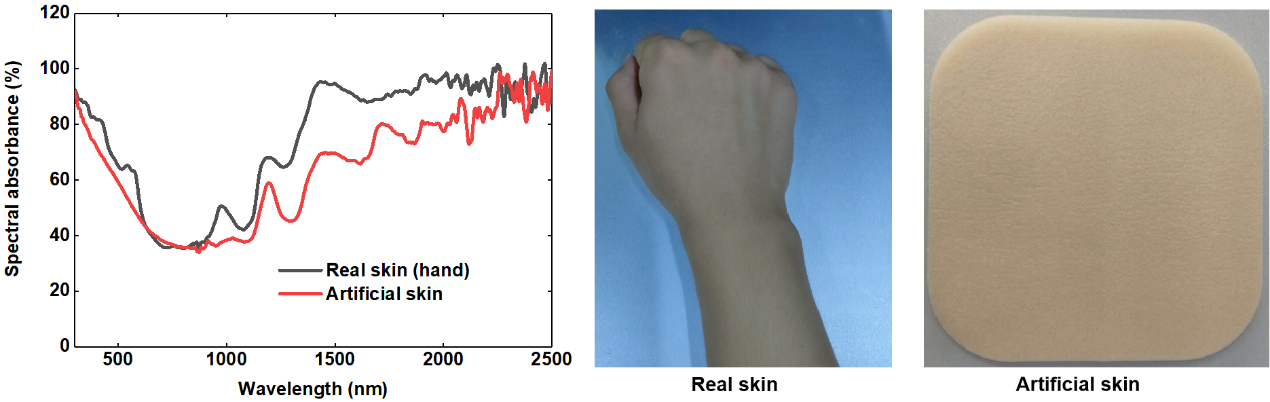


**Figure S10** Comparison of the spectral absorption of the Real skin and Artificial skin.


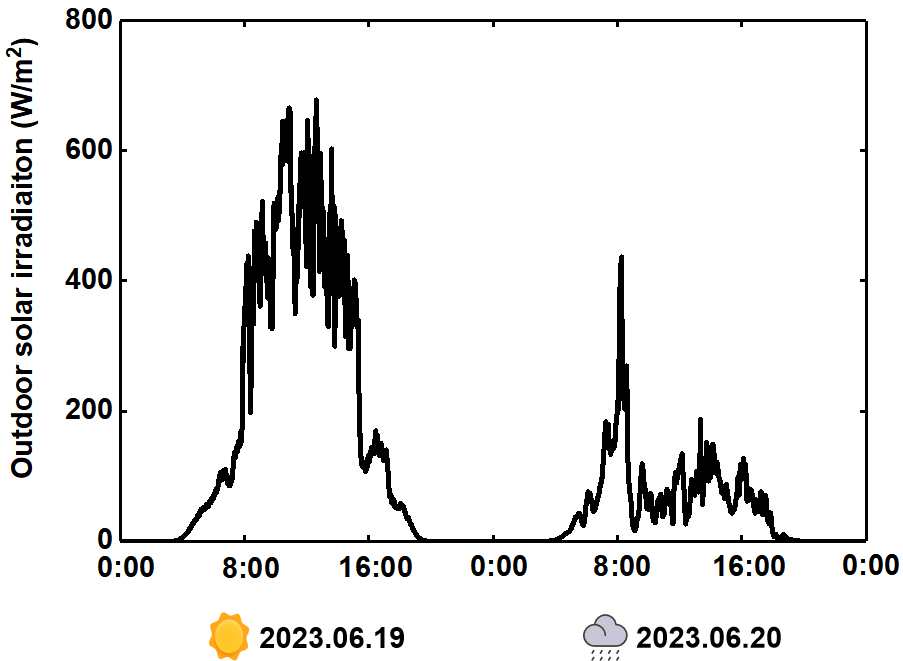


**Figure S11** Outdoor solar irradiance of June 19 and June 20, 2023.


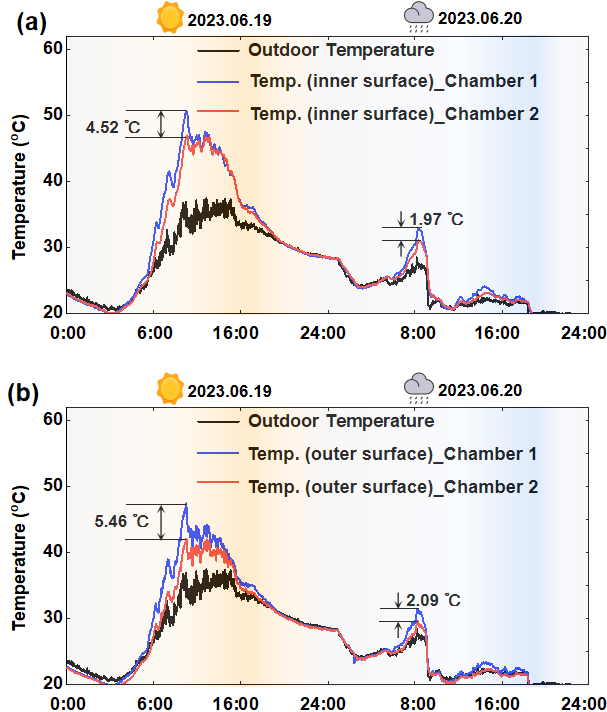


**Figure S12** Comparisons of the surface temperature of the RB windows in Chamber 1 and Chamber 2.

**Figure S13** Annual space heating and cooling energy-saving of the RB window and double-layered low-e window in different cities.


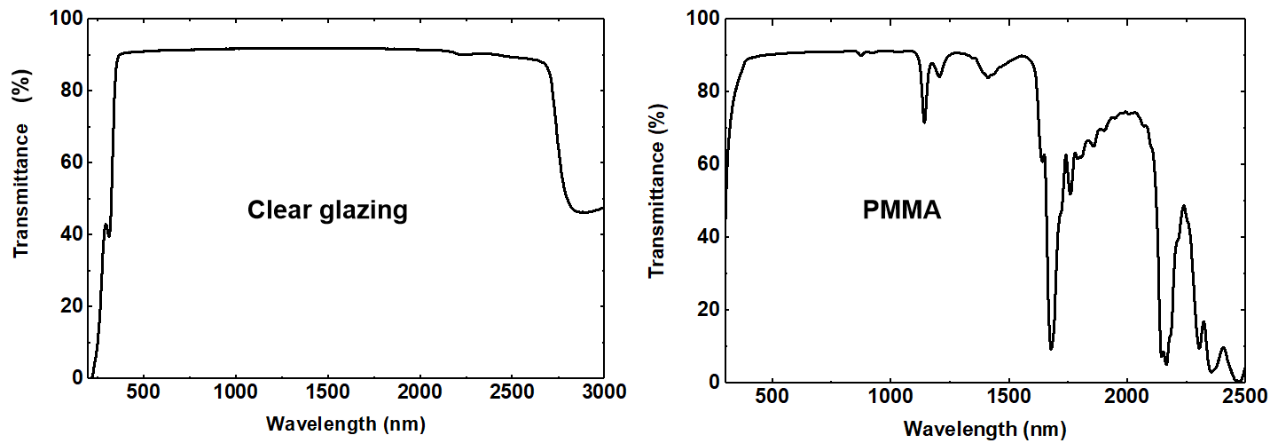


**Figure S14** Spectral transmittance of clear glazing and PMMA pane used in this study

**Table S1**. Categories, materials, luminous transmittance and solar modulation ability of the thermochromic (***TC***) and electrochromic (***EC***) smart windows

| **Categories** | ***TC* materials (*EC* materials)** | | ***T*_lum_ (%)** | | **Δ*T*_sol_ (%)** | **Ref** |
| --- | --- | --- | --- | --- | --- | --- |
|  |  |  | **Bleached** | **Opaque** |  |  |
| VO_2_-based ***TC*** windows | Signal layer VO_2_ | | 78 | 77 | 5.9 | Wang *et al.* 2015 ^33^ |
|  |  |  | 42.6 | 39.7 | 15.5 | Xiang *et al.* 2022 ^34^ |
|  |  |  | 56.4 | 55.6 | 12.2 |  |
|  | Multi-layered VO_2_ | Si-Al/VO_2_ | 46.3 | 41.4 | 18.9 | Liu *et al.* 2018 ^16^ |
|  |  | TEOS/VO_2_ | 52.7 | 48.7 | 16.4 |  |
|  |  | VO_2_(55nm)/TiO_2_(140nm) | 50.6 | 52.1 | 7.2 | Chen *et al.* 2011 ^35^ |
|  |  | VO_2_(55nm)/TiO_2_(420nm) | 58 | 53.9 | 10.9 |  |
|  |  | VO_2_(90nm)/SiO_2_(85nm) | 70.1 | 66.2 | 11.6 | Liu *et al.* 2020 ^36^ |
|  | Zn doped VO_2_ | | 47.2 | 51.92 | 12.1 | Kang *et al.* 2021 ^37^ |
|  | Si doped VO_2_ | | 54.7 | | 13.9 | Zou *et al.* 2021 ^38^ |
|  | Zr doped VO_2_ | | 60.4 | | 14 | Shen *et al.* 2014 ^39^ |
|  | Ti doped VO_2_ | | 53 | | 17.2 | Chen *et al.* 2013 ^39^ |
|  | VO_2_-based nanocomposites | | 52.9 | 37.3 | 25.7 | Zhao *et al*. 2023 ^19^ |
|  |  |  | 63.7 | 54.4 | 12 | Liu *et al.* 2014 ^41^ |
|  |  |  | 43.3 | 39.9 | 14.1 |  |
|  |  |  | ～50 | ～50 | 17 | Zhao *et al.* 2020 ^42^ |
|  |  |  | ～70 | ～60 | 20 | Zhao *et al.* 2020 ^43^ |
| Hydrogel-based ***TC*** window |  | | 89.1 | 0.6 | 73.9 | Zhou *et al.* 2013 ^44^ |
|  |  | | 91.8 | 73.7 | 73.7 | Zhang *et al.* 2022 ^23^ |
|  |  | |  |  |  |  |
|  |  | | 87.2 | 0.5 | 81.6 | Li *et al.* 2019 ^21^ |
|  |  | | 90.2 | 39 | 42.1 | Zhou *et al.* 2020 ^20^ |
|  |  | | 90.9 | 1.26 | 68.1 |  |
| VO_2_/hydrogel hybrid products | | | 86.8 | 61.8 | 25 | Zhou *et al.* 2015 ^10^ |
|  |  |  | 82.1 | 43.2 | 38.9 |  |
| ***EC*** window | WO_3-_*_x_* nanoflowers | | 69.7 | 41.7 | 43.6 | Huang *et al.* 2023^45^ |
|  | WO*_x_*-based plasmochromic | | 79 |  | 70 | Cots *et al.* 2021 ^26^ |
|  | Ta-doped TiO_2_ nanocrystals | | 93.4 | 4.3 | 80 | Cao *et al.* 2019 ^27^ |
|  | WO_3-_*_x_*-NbO*_x_* nanocomposite | | 93 | 22 | 78 | Kin *et al.* 2015 ^47^ |
| ***EC/TC*** dual-responsive windows | H^+^ doped hydrogel-WO_3_ | thermochromic | 78.5 | 22.3 | 42.4 | Zhang *et al.* 2023^48^ |
|  |  | electrochromic | 78.5 | 16.3 | 49.3 |  |
|  |  | thermo-/electro- dual-chromic | 78.5 | 4 | 57.2 |  |
|  | Li^+^ doped hydrogel-WO_3_ | thermochromic | 75.9 | 12 | 46.5 |  |
|  |  | electrochromic | 75.9 | 22.1 | 44.3 |  |
|  |  | thermo-/electro- dual-chromic | 75.9 | 4.1 | 54.9 |  |
| ***PC*** | Cu-W-PC |  | 91 | 18 | 73 | Meng *et al.* 2023^7^ |
| ***RB* window** | Bio-mass based dyes | 2 mm | 90.2 | 66.4 | 51.7 | this work |
|  |  | 3 mm | 90.4 | 56.2 | 60.6 |  |
|  |  | 5 mm | 89.7 | 41.0 | 67.6 |  |
|  |  | 10 mm | 89.4 | 19.4 | 74.6 |  |

**Table S2** Comparison of the bio-massed SSF and other green dyes used in spectrally selective windows

| ***Dyes*** | ***Color*** | Tsol | Tlum | ***T_lum_/T_sol_*** | ***CRI*** | ***Color coordinates*** | | **Refs.** |
| --- | --- | --- | --- | --- | --- | --- | --- | --- |
|  |  |  |  |  |  | ***x*** | ***y*** |  |
| 0.1wt% F_2_O_3_ doped in reducing atmosphere | Green | 0.707 | 0.903 | 1.28 | 97 | 0.333 | 0.346 | *Fu et al.****^3^*** |
| 0.2wt% F_2_O_3_ doped in reducing atmosphere | Green | 0.667 | 0.888 | 1.33 | 97 | 0.334 | 0.346 |  |
| 0.3wt% F_2_O_3_ doped in reducing atmosphere | Green | 0.637 | 0.885 | 1.39 | 97 | 0.334 | 0.346 |  |
| 0.4wt% F_2_O_3_ doped in reducing atmosphere | Green | 0.476 | 0.709 | 1.49 | 96 | 0.337 | 0.348 |  |
| 0.62wt% Fe_2_O_3_, 0.12wt% FeO, 0.1wt% TiO_2_, 7ppm CoO | Green | 0.525 | 0.777 | 1.48 | 95 | 0.325 | 0.352 | *Central Glass, Inc.****^4^*** |
| 0.4wt% Fe_2_O_3_, 1.1wt% CeO_2_, 1.1wt% TiO_2_, 17ppm Cr_2_O_3_, 0.2wt% FeO | Green | 0.389 | 0.736 | 1.89 | 87 | 0.326 | 0.375 |  |
| 0.54wt% Fe_2_O_3_, 0.13wt%FeO, 0.1wt% TiO_2_, 17ppm Cr_2_O_3_ | Green-blue | 0.539 | 0.776 | 1.44 | 96 | 0.325 | 0.351 |  |
| 0.38wt% Fe_2_O_3_, 1wt% CeO_2_, 1wt% TiO_2_, 0.5wt% SnO, 17ppm Cr_2_O_3_, 0.17wt% FeO | Green-blue | 0.432 | 0.706 | 1.63 | 92 | 0.328 | 0.362 |  |
| 0.54wt% Fe_2_O_3_, 0.14wt% FeO, 0.24wt% SO_3_ | Green | 0.515 | 0.780 | 1.51 | 94 | 0.322 | 0.351 | *Pecoraro et al.****^5^*** |
| 0.74wt% Fe_2_O_3_, 0.196wt% FeO, 0.22wt% SO_3_ | Green-blue | 0.413 | 0.667 | 1.62 | 92 | 0.316 | 0.350 |  |
| 0.80wt% Fe_2_O_3_, 0.23wt% FeO, 0.03wt% SO_3_ | Green-blue | 0.378 | 0.672 | 1.78 | 90 | 0.314 | 0.353 |  |
| 0.1% SC/10% CuSO_4_ with thickness of 2 mm | Green | 0.345 | 0.664 | 1.92 | 85 | 0.31 | 0.37 | ***This work*** |
| 0.1% SC/10% CuSO_4_ with thickness of 3 mm | Green | 0.247 | 0.562 | 2.28 | 80 | 0.31 | 0.38 |  |
| 0.1% SC/10% CuSO_4_ with thickness of 5 mm | Green | 0.151 | 0.41 | 2.72 | 68 | 0.29 | 0.4 |  |

**Table S3**. Detailed optical properties of the APPs window in cool and hot state, with different spectral splitting fluids

| **Thickness** | **Concentration** | ***T*_lum_ (%)** | | ***T*_sol_ (%)** | | **Δ*T*_sol_ (%)** | **SHGC** | | ***T*_mel_ (%)** | | ***LSG*** | ***CAF*_colored_** | ***CRI*_colored_** | **Color coordinates** | |
| --- | --- | --- | --- | --- | --- | --- | --- | --- | --- | --- | --- | --- | --- | --- | --- |
|  |  | **Bleached** | **Colored** | **Bleached** | **Colored** |  | **Bleached** | **Colored** | **Bleached** | **Colored** |  |  |  | ***x*** | ***y*** |
| 2 mm | 20% CuSO_4_ | 90.2 | 74.6 | 86.2 | 36.5 | 47.0 | 0.74 | 0.40 | 89.8 | 85.5 | 1.86 | 1.15 | 83 | 0.29 | 0.34 |
|  | 10% CuSO_4_ | 90.2 | 80.7 | 86.2 | 46.2 | 37.3 | 0.74 | 0.47 | 89.8 | 86.7 | 1.73 | 1.07 | 92 | 0.31 | 0.34 |
|  | 5% CuSO_4_ | 90.2 | 84.0 | 86.2 | 57.7 | 25.8 | 0.74 | 0.55 | 89.8 | 86.8 | 1.54 | 1.03 | 97 | 0.32 | 0.34 |
|  | 0.15% SC | 90.2 | 64.6 | 86.2 | 60.2 | 23.3 | 0.74 | 0.56 | 89.8 | 61.5 | 1.15 | 0.95 | 91 | 0.34 | 0.38 |
|  | 0.10% SC | 90.2 | 70.7 | 86.2 | 63.1 | 20.4 | 0.74 | 0.58 | 89.8 | 69.1 | 1.21 | 0.98 | 92 | 0.33 | 0.37 |
|  | 0.05% SC | 90.2 | 79.9 | 86.2 | 69.2 | 14.3 | 0.74 | 0.62 | 89.8 | 78.5 | 1.28 | 1.08 | 96 | 0.33 | 0.36 |
|  | 10% CuSO_4_ | 90.2 | 84 | 86.2 | 45.9 | 40.3 | 0.74 | 0.47 | 89.8 | 90.1 | 1.80 | 1.153 | 91 | 0.31 | 0.34 |
|  | 0.05% SC/10% CuSO_4_ | 90.2 | 74.5 | 86.2 | 41.1 | 45.1 | 0.74 | 0.43 | 89.8 | 74.3 | 1.72 | 0.997 | 89 | 0.32 | 0.36 |
|  | 0.1% SC/10% CuSO_4_ | 90.2 | 66.4 | 86.2 | 34.5 | 51.7 | 0.74 | 0.39 | 89.8 | 63.4 | 1.71 | 0.968 | 85 | 0.31 | 0.37 |
| 3 mm | 10% CuSO_4_ | 90.4 | 81 | 85.3 | 39.3 | 46 | 0.73 | 0.42 | 90 | 90.4 | 1.93 | 1.116 | 88 | 0.3 | 0.34 |
|  | 0.05% SC/10% CuSO_4_ | 90.4 | 74.1 | 85.3 | 35.6 | 49.7 | 0.73 | 0.40 | 90 | 73.6 | 1.87 | 0.993 | 84 | 0.31 | 0.37 |
|  | 0.1% SC/10% CuSO_4_ | 90.4 | 56.2 | 85.3 | 24.7 | 60.6 | 0.73 | 0.32 | 90 | 54.1 | 1.75 | 0.964 | 80 | 0.31 | 0.38 |
| 5 mm | 10% CuSO_4_ | 89.7 | 78.4 | 82.7 | 33.7 | 49 | 0.72 | 0.38 | 89.6 | 89.4 | 2.05 | 1.195 | 81 | 0.28 | 0.34 |
|  | 0.05% SC/10% CuSO_4_ | 89.7 | 55.9 | 82.7 | 21.9 | 60.8 | 0.72 | 0.30 | 89.6 | 55 | 1.85 | 0.983 | 72 | 0.30 | 0.38 |
|  | 0.1% SC/10% CuSO_4_ | 89.7 | 41 | 82.7 | 15.1 | 67.6 | 0.72 | 0.26 | 89.6 | 39.3 | 1.60 | 0.959 | 68 | 0.29 | 0.40 |
| 10 mm | 10% CuSO_4_ | 89.4 | 65.1 | 80.6 | 29.5 | 61.1 | 0.70 | 0.35 | 88.7 | 88.8 | 1.84 | 1.364 | 68 | 0.25 | 0.33 |
|  | 0.05% SC/10% CuSO_4_ | 89.4 | 38.6 | 80.6 | 12.6 | 68 | 0.70 | 0.24 | 88.7 | 37.2 | 1.61 | 0.964 | 56 | 0.27 | 0.41 |
|  | 0.1% SC/10% CuSO_4_ | 89.4 | 19.4 | 80.6 | 6.0 | 74.6 | 0.70 | 0.19 | 88.7 | 19 | 1.00 | 0.977 | 49 | 0.26 | 0.43 |

Notes: (1) The SHGC values were calculated according to Equations 3 to 5, as depicted in the supporting information.

**Table S4.** Detailed information of the clear glazing, low-E glazing, and RB window used in the simulation.

|  | Double layered clear window | Double layered Low-e window | RB window in bleached state | RB window in colored state |
| --- | --- | --- | --- | --- |
| U-factor | 1.361 | 1.224 | 1.404 | 1.435 |
| SHGC | 0.817 | 0.627 | 0.821 | 0.312 |

Notes: (1) Double glazing in the simulation work is made of two clear glazing with air gap of 13 mm;

1. Double layered low-e glazing is made of a low-e glazing and a clear glazing, with air gap of 13mm;

**References**

1. Energy Performance Ratings for Windows, Doors, and Skylights. <https://www.energy.gov/energysaver/energy-performance-ratings-windows-doors-and-skylights.>
2. J. Oh, S. Yang, Y. Do. Healthy, natural, efficient and tunable lighting: four-package white LEDs for optimizing the circadian effect, color quality and vision performance, Light: Science & Applications, 3 (2014) e141.
3. L. Fu, W. Ren, C. Liu, S. Xu, R. Zheng, W. Wei, C. Zhang, B. Peng. Spectroscopic and laser-induced damage properties of Fe^2+^-doped fluorophosphate glass, a new color-separation material. Applied Physics B, 113 (2013) 81-86.
4. Taishi Taguchi and Yoshiko miyazaka, Ultraviolet and infrared absorbing green glass (in Chinese). Patent number: CN1874967 A.
5. G..Pecoraro and L. Shelestak, Transparent Infrared Absorbing Glass and Method of Making, Patent number: 4792536.
